# A Modified Eco-Efficiency Framework and Methodology for Advancing the State of Practice of Sustainability Analysis as Applied to Green Infrastructure

**DOI:** 10.1002/ieam.1928

**Published:** 2017-04-19

**Authors:** Santosh R Ghimire, John M Johnston

**Affiliations:** † Former Oak Ridge Institute for Science and Education (ORISE) Postdoctoral Research Participant, US Environmental Protection Agency, Office of Research and Development, Athens, Georgia; ‡ US Environmental Protection Agency, Office of Research and Development, Athens, Georgia

**Keywords:** Data Envelopment Analysis, Modified eco-efficiency framework, Green infrastructure, Sustainability Rainwater harvesting

## Abstract

We propose a modified eco-efficiency (EE) framework and novel sustainability analysis methodology for green infrastructure (GI) practices used in water resource management. Green infrastructure practices such as rainwater harvesting (RWH), rain gardens, porous pavements, and green roofs are emerging as viable strategies for climate change adaptation. The modified framework includes 4 economic, 11 environmental, and 3 social indicators. Using 6 indicators from the framework, at least 1 from each dimension of sustainability, we demonstrate the methodology to analyze RWH designs. We use life cycle assessment and life cycle cost assessment to calculate the sustainability indicators of 20 design configurations as Decision Management Objectives (DMOs). Five DMOs emerged as relatively more sustainable along the EE analysis Tradeoff Line, and we used Data Envelopment Analysis (DEA), a widely applied statistical approach, to quantify the modified EE measures as DMO sustainability scores. We also addressed the subjectivity and sensitivity analysis requirements of sustainability analysis, and we evaluated the performance of 10 weighting schemes that included classical DEA, equal weights, National Institute of Standards and Technology’s stakeholder panel, Eco-Indicator 99, Sustainable Society Foundation’s Sustainable Society Index, and 5 derived schemes. We improved upon classical DEA by applying the weighting schemes to identify sustainability scores that ranged from 0.18 to 1.0, avoiding the nonuniqueness problem and revealing the least to most sustainable DMOs. Our methodology provides a more comprehensive view of water resource management and is generally applicable to GI and industrial, environmental, and engineered systems to explore the sustainability space of alternative design configurations.

## INTRODUCTION

Sustainable water resource management faces many challenges. Urbanization and intensification of agriculture are the most important anthropogenic land cover changes affecting hydrologic systems and biodiversity (Kalnay and Cai 2003; Seto et al. 2012; Ball et al. 2013; Dubé et al. 2013). Drought and climate change also affect the timing and amount of water supplies (IPCC 2008; Immerzeel et al. 2010), and rising global population combined with increased urbanization magnifies water resource demands worldwide (Vörösmarty et al. 2000; UN 2011; Hoekstra and Wiedmann 2014; Schewe et al. 2014). Green infrastructure (GI) uses natural processes, landform, soils, and vegetation to meet water resource needs. It includes rainwater harvesting (RWH), green roofs, rain gardens, permeable pavements, and vegetated swales. Green infrastructure also holds promise for mitigating climate change, improving human and ecological health, and increasing water and energy efficiency (EEA 2011; USEPA 2013a). The US Environmental Protection Agency (USEPA) is focusing on GIs to meet water resource challenges in its GI Action Strategy (USEPA 1996, 2008). Rainwater harvesting, a technique of capturing and storing rainwater, provides benefits that are recognized globally (USEPA 2008; EEA 2011; Ghimire and Johnston 2015). The benefits of RWH include cost and water savings (Ghisi et al. 2009; Ghimire et al. 2012; Belmeziti et al. 2013) and life cycle environmental and human health impacts (Ghimire et al. 2014; Wang and Zimmerman 2015). Lack of data on GI efficacy and its environmental and human health impacts hinders informed decision making in watershed management (NRC 2009). Integrating environmental, social, and economic impacts in a single sustainability score remains a challenge.

Sustainability of a system or society involves a long-term interplay of economic, social, and environmental goals, which are in turn complicated by the complex, dynamic, and interdependent nature (across scales and temporal and spatial variation) of natural, man made, and social systems (Fiksel 2006; Fiksel et al. 2009; Hecht et al. 2014; Liu et al. 2015). According to Allen et al. (2003), sustainability seeks answers to 4 questions: what to sustain, for whom, for how long, and at what cost? The value of a sustainable system is subject to stakeholders’ values and agreements and is thus a “wicked” problem: It is not “true-or-false” but a “good-or-bad” answer (Rittel and Webber 1973). Although life cycle assessment (LCA) (Hendrickson et al. 2006; Koehler 2008; Finnveden et al. 2009), life cycle cost assessment (LCCA) (Fuller and Petersen 1996; Ghimire et al. 2012), and social LCA (Jørgensen et al. 2010) methods have been used in reporting environmental, economic, and social dimensions of sustainability, a more comprehensive method that integrates each is evolving in recent years (Guinee et al. 2011). However, integration of the multidisciplinary indicators remains a challenge.

Originating in the business sector, eco-efficiency (EE) has become a popular approach to quantify environmental sustainability. The term “EE” was first used by Schaltegger and Sturm (1989), then publicized by the World Business Council for Sustainable Development (WBCSD) at the United Nations Conference on Environment and Development during the Rio Earth Summit in 1992 (Lehni et al. 2000). Our literature review suggested widespread application of EE in business, chemical manufacture, building construction, and road transportation sectors with varying definitions and selection criteria of EE indicators (see Supplemental Data). Although the utility of EE has been demonstrated, much less attention has been devoted to water resource management. Despite broad application, critics take issue with the limitation of the rebound effect phenomenon in EE, that is, an increase in EE leading to an increase in material and/or energy use (Bjørn and Hauschild 2013). Much of the literature utilized economic indicators of life-cycle costs, gross domestic product, net income, and value added. Environmental indicators considered include global warming, energy use, climate change, acidification, eutrophication, ecotoxicity, human health, and water use. Most studies used LCA and LCCA to calculate EE indicators and applied Data Envelopment Analysis (DEA); only a few considered social indicators such as number of accidents, wages and salaries, human rights, crimes, and corruptions (Jørgensen et al. 2008). The social value of any system depends on stakeholders’ interests and priorities, so the amount and selection of indicators vary by necessity. We propose using a modified EE framework and sustainability assessment methodology that integrates environmental, economic, and social indicators into a single measure, consistent with the state of practice of sustainability. The approach supports objectives of the USEPA’s GI Action Strategy (2008) and is consistent with the US Green Building Council’s Leadership in Energy and Environmental Design (LEED) (USGBC 2013) rating system to evaluate environmental and human health impacts.

Life cycle assessment and LCCA are accepted ways to calculate EE indicators avoiding problem-shifting effects (Brattebø 2005; Gabriel and Braune 2005). Life cycle assessment avoids these effects by minimizing life cycle impacts at one location or stage, while also avoiding the impacts elsewhere or at another stage of a system or service (USEPA 2006). Life cycle assessment is consistent with the International Organization for Standardization (ISO) guidelines for quantifying material and energy flow and environmental impacts in a cradle-to-grave approach (ISO 2006a; 2006b), whereas LCCA emphasizes life cycle costs and discounting of future costs (Fuller and Petersen 1996).

In EE analysis, weighting and aggregation of impacts is controversial (Wu et al. 2012), including the subjectivity of expert opinion. Therefore, it is important that subjectivity be explicitly documented. Existing weighting methods include expert judgment approaches such as Eco-Indicator 99 (EI99) (Goedkoop and Spriensma 2000), equal and expert judgment weights to the Sustainable Society Index scheme (SSIS) (Sironen et al. 2014), the National Institute of Standards and Technology (NIST) stakeholder panel (Gloria et al. 2007), and statistical approaches such as classical DEA (CDEA) (Seppälä and Hämäläinen 2001). Eco-Indicator 99 (EI99) aggregates environmental impacts in a single score, assigning different weighting schemes to damage categories of Human Health (40%), Ecosystem Quality (40%), and Resources (20%), based on written responses to a questionnaire (Goedkoop and Spriensma 2000). The SSIS measures the human, environmental, and economic sustainability of 151 countries by considering Human well-being (39%), Environmental well-being (36%), and Economic well-being (25%) (Sironen et al. 2014). The NIST weights were determined on the basis of panel voting, which set global warming at the highest weight at 29%, followed by fossil fuel depletion at 10%, and all others in single-digit impact importance (Gloria et al. 2007).

Data Envelopment Analysis is the most widely applied statistical approach in EE analysis, used for both weighting and aggregating environmental and economic indicators. Pioneered by Farrell (1957) and Charnes et al. (1978), it was examined by many others, for example, Kuosmanen and Kortelainen (2005) in EE analysis of road transportation. Despite its advantages, CDEA has limitations of data size and nonhomogeneity, EE nonuniqueness, and relative assessment (Dyson et al. 2001; Zhang et al. 2008; Sironen et al. 2014). Eco-efficiency nonuniqueness, producing more than 1 optimal solution, is a main limitation of CDEA (Sironen et al. 2014). “Relative assessment” refers to the absence of an absolute or optimal solution because products or systems are evaluated in relationship to each other. Nonhomogeneous data include outliers and dimensionality issues when integrating values across orders of magnitude. One of the methods to increase data homogeneity in DEA is mean normalization (Sarkis 2007).

### Objective and novelty

Our objective is to demonstrate the use of a modified EE framework within a novel sustainability analysis methodology for various RWH design configurations as Decision Management Objectives (DMOs), minimizing the limitations of CDEA and eliminating the EE nonuniqueness problem. We utilize LCA and LCCA to calculate sustainability indicators and DEA to calculate the modified EE measures as sustainability scores. The indicators consist of 1 economic indicator (life cycle cost), 4 environmental indicators (blue water use, ecotoxicity, cumulative energy demand, and global warming potential), and 1 social indicator (human health cancer impacts). We also address the performance of 10 weighting schemes, 5 existing weighting schemes (EI99, equal weights [EQWT], SSIS, NIST, and CDEA) and 5 derived schemes based on impact thresholds. Our methodology considered at least 1 indicator from each dimension of sustainability and weighting schemes with equal and unequal thresholds consistent with best practice. To our knowledge, no other study has combined these methods to analyze GI sustainability, and the methodology is applicable to other environmental, industrial, and engineered systems. In the following sections, we describe the modified EE framework and demonstrate the methodology.

## METHODS

### Modified EE framework

#### Definition of EE indicators

We define an EE indicator as a quantity that describes the state or level of a process or system suitable for EE measurement. An EE measure is defined as the ratio of economic output to environmental input of a process or system (Eqn. 1). We set the EE measure as the ratio of the economic and environmental indicators. This approach is consistent with the definition used by Lehni et al. (2000) and is similar to the definition of technical efficiency as the ratio of technical output to input (Farrell 1957). We further modified the EE measure by including a social indicator in addition to the traditional environmental indicator (Eqn. 2).

(1) $$E=I_{\text{ECO}}/I_{\text{ENV}},$$

(2) $$E_{\mathrm{mod}}=I_{\text{ECO}}/I_{\text{SOC}},$$

where *E* = traditional EE measure; *E*_mod_ = modified EE measure; *I*_ECO_ = economic indicator, for all *A_i_* (economic value, output); *I*_ENV_ = environmental indicator, for all *D_i_* (environmental impact, input); and *I*_SOC_ = social indicator, for all *S_i_* (social impact).

The modified EE framework consists of 18 indicators synthesized from Ghimire et al. (2012), USEPA (2012), and Ghimire and Johnston (2013) (Table 1). Within the context of the modified framework (Table 1), the traditional EE measure is defined as the ratio of the economic indicator, for example, life cycle costs (present value, *A*_LC_), to environmental indicator, energy demand (*D*_ED_):

(3) $$E=I_{\text{ECO}}/I_{\text{ENV}}=A_{\text{LC}}/D_{\text{ED}}.$$

And a modified EE measure is defined by integrating the social indicator, for example, total human health toxicity (cancer, *S*_HH_) with *A*_LC_:

(4) $$E_{\mathrm{mod}}=I_{\text{ECO}}/I_{\text{SOC}}=A_{\text{LC}}/S_{\text{HH}}.$$

#### Selection of indicators

There is no universal suite of sustainability metrics (McCormick et al. 2013; Stahl and Bridges 2013). The number and type of indicators vary by research focus and sector: Examples from the transportation and manufacturing sectors range from 3 environmental indicators (greenhouse gas [GHG] emissions, energy use, and water withdrawals) to 5 environmental indicators to 10 environmental indicators and 1 economic indicator (Kuosmanen and Kortelainen 2005; Kortelainen and Kuosmanen 2007; Leal et al. 2012; Tatari and Kucukvar 2012). We selected at least 1 indicator from each dimension of sustainability consistent with Guinee et al. (2011) for a total of 6 to demonstrate the methodology. Furthermore, selected indicators are simple and acceptable as used in previous EE analysis criteria (UNCTAD 2004; Brattebø 2005; Kielenniva et al. 2012) and LCA standards of the ISO (ISO 2006a, 2006b).

### Sustainability analysis using modified EE framework

#### Life cycle assessment and LCCA

The OpenLCA tool was utilized for LCA calculations in conjunction with TRACI 2.0, the USEPA’s Tool for the Reduction and Assessment of Chemical and Other Environmental Impacts, and ReCiPe method (Goedkoop et al. 2012; OpenLCA 2013; USEPA 2013b; Ghimire et al. 2014). We performed LCCA following the US Department of Energy’s LCCA handbook (Fuller and Petersen 1996), the US guidelines for LCCA (Greening the Government…1999, Sect. 707, p 30860), and other literature (Ghimire et al. 2012; NIST 2013). Life cycle assessment and LCCA data were normalized using the mean-normalization method in DEA formulation (Sarkis 2007). A description of LCCA, LCA data, and data normalization is provided in Supplemental Data.

#### Data Envelopment Analysis calculation of modified EE measure to produce sustainability score

Our methodology requires a linear integration of multiple indicators, and DEA facilitated integration of environmental, economic, and social indicators (Eqn. 5, 5a–5d; Eqn. 6, 6a–6d). The CDEA optimization begins with standard EE as the economic output divided by the linear function of environmental input (Kuosmanen and Kortelainen 2005). The *n*th DMO of *N* DMOs induces *X* environmental impacts measured by *D_nX_*. Each DMO has 1 economic indicator, *A_n_*.

(5) $$\text{Maximize}\;E_{n}=\frac{A_{n}}{w_{1}D_{n1}+w_{2}D_{n2}+....+w_{X}D_{nX}}\;\;\;(\text{for}\;\text{all}\;n=1\;\text{to}\;N),$$

subject to

(5a) $$\frac{A_{1}}{w_{1}D_{11}+w_{2}D_{12}+....+w_{X}D_{1X}}\leq 1,$$

(5b) $$\frac{A_{2}}{w_{1}D_{21}+w_{2}D_{22}+....+w_{X}D_{2X}}\leq 1,$$

(5c) $$\frac{A_{N}}{w_{1}D_{N1}+w_{2}D_{N2}+....+w_{X}D_{NX}}\leq 1,$$

(5d) $$w_{1},w_{2},\ldots w_{X}\geq 0,$$

where *E* = modified EE measure or sustainability score; *A* = economic indicator; *D* = environmental indicator; *w_i_* = model weight estimated by DEA optimization; and *i* ranges from 1 to *X*, the number of environmental and social impacts (in this example, *X* = 5).

A random number is generated between 0 to 1 as an initial value of each *w_i_*, which is then optimized by DEA. Equation 5 and the restrictions (Eqn. 5a–5d) are nonlinear functions and must be transformed to linear form by determining the inverse functions (Eqn. 6 and 6a–6d):

(6) $$\text{Maximize}\;{E^{-1}}_{n}=\frac{w_{1}D_{n1}}{A_{n}}+\frac{w_{2}D_{n2}}{A_{n}}+\ldots +\frac{w_{X}D_{nX}}{A_{n}},$$

subject to

(6a) $$\frac{w_{1}D_{11}}{A_{1}}+\frac{w_{2}D_{12}}{A_{1}}+.....+\frac{w_{X}D_{1X}}{A_{1}}\geq 1,$$

(6b) $$\frac{w_{1}D_{21}}{A_{2}}+\frac{w_{2}D_{22}}{A_{2}}+\ldots +\frac{w_{X}D_{2X}}{A_{2}}\geq 1,$$

(6c) $$\frac{w_{1}D_{n1}}{A_{n}}+\frac{w_{2}D_{n2}}{A_{n}}+\ldots +\frac{w_{X}D_{nX}}{A_{n}}\geq 1,$$

(6d) $$w_{1},\;w_{2},\ldots w_{X}\geq 0.$$

The CDEA formulation is solved for each DMO using Excel Solver, and the modified EE measures (or sustainability scores) are estimated by calculating the inverse of the optimal scores. Each solution also produces optimized weights for each environmental and social indicator. Data Envelopment Analysis optimizes weights and sustainability scores subject to the imposed weighting restrictions.

#### Sensitivity analysis of weighting schemes using improved DEA

The 10 weighting schemes evaluated are CDEA, EQWT, NIST, EI99, SSIS, Threshold 1 to SSIS (*w*_1_ = *w*_5_ and *w*_2_ = *w*_4_), Threshold 2 to SSIS (*w*_1_:*w*_5_ = 67%:33%; *w*_2_:*w*_4_ = 67%:33%), Threshold 3 to SSIS (*w*_1_:*w*_5_ = 33%:67%; *w*_2_:*w*_4_ = 33%:67%), Threshold 4 to EI99 (*w*_1_:*w*_5_ = 67%:33%; *w*_2_:*w*_4_ = 67%:33%), and Threshold 5 to EI99 (*w*_1_: *w*_5_ = 33%:67%; *w*_2_:*w*_4_ = 33%:67%); *w*_1_, *w*_2_, *w*_3_, *w*_4_, and *w*_5_ being the weights of blue water use, ecotoxicity, cumulative energy demand, global warming potential, and human health cancer impacts, respectively. We imposed EQWT to all impacts, equal weights to impacts of Human well-being and Environmental well-being categories of SSIS (Threshold 1 to SSIS), and unequal thresholds to SSIS (Thresholds 2 and 3) and EI99 (Thresholds 4 and 5) (Table 3).

Classical DEA without expert judgment restrictions (Eqn. 6 and 6a–6d) was improved by incorporating additional expert judgment restrictions necessary for each of the weighting schemes. Details on all weighting schemes and necessary constraints are provided in Supplemental Data. The improved DEA formulation of each weighting scheme is solved for each DMO, and sustainability scores are estimated. Each solution also produced optimized weights for each environmental and social indicator.

We note that additional inequality constraints such as subjective valuation of environmental damages may be imposed if weights of environmental damage differ across indicators. There may be a large number of expert judgment weighting schemes, depending on the number of experts and environmental and social indicators of interest. Further, each indicator can have a large number of weights (*w_i_* from 0 to 1).

### Sustainability analysis of RWH example

We demonstrated the methodology using the domestic RWH design from Ghimire et al. (2014). The 20 DMO options of the domestic RWH system differed by materials (plastic pipe, cast iron pipe, polyethylene tank, and concrete tank), energy use (with and without pump), and energy type (low-and medium-voltage electricity and photovoltaic) (details in Supplemental Data). First, we selected 4 environmental, 1 economic, and 1 social indicator from the modified EE framework. These are cumulative energy demand, global warming potential, ecotoxicity, blue water use, human health, and life cycle cost.

**Figure 1:**
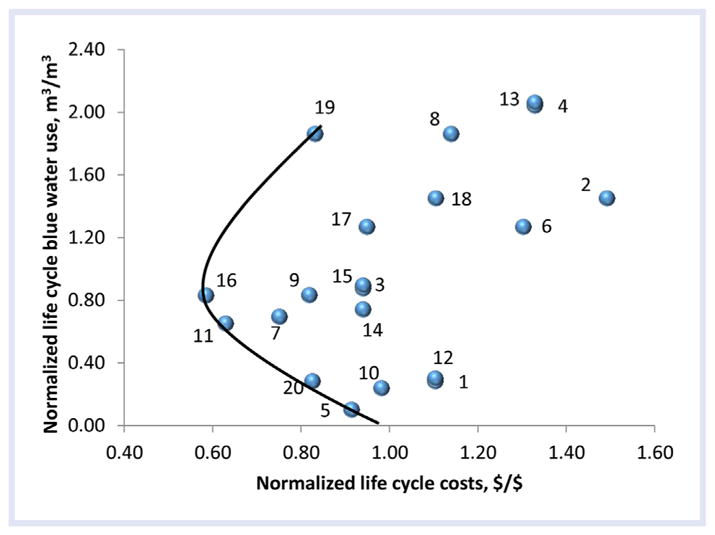
Eco-efficiency plot for 20 DMOs suggesting a Tradeoff Line with a potential sustainable solution domain. DMO = decision management objective.

Second, we conducted LCA and LCCA to calculate the indicators of the 20 DMOs. The mean-normalized life-cycle blue water use and life-cycle cost analysis provided a subset of 5 relatively more sustainable DMO options along the Tradeoff Line (Figure 1, Table 2). A complete description of all 20 DMOs, the mean-normalization method, and normalized data are provided in Supplemental Data. We used CDEA without expert judgment constraints (Eqn. 6 and 6a–6d) to integrate the environmental, social, and economic indicators of the 5 DMOs. The CDEA formulation of DMO5 is provided (Eqn. 7–13); all other DMOs follow the same form, with the corresponding mean-normalized values associated with each of the weights, *w_i_* (Table 2):

(7) $$\mathrm{Minimize}\;{E^{-1}}_{n}=\frac{1}{0.91}[0.10w_{1}+0.49w_{2}+0.52w_{3}+0.50w_{4}+0.27w_{5}],$$

subject to

(8) $$\frac{1}{0.91}[0.10w_{1}+0.49w_{2}+0.52w_{3}+0.50w_{4}+0.27w_{5}]\geq 1,$$

(9) $$\frac{1}{0.91}[0.65w_{1}+0.17w_{2}+0.20w_{3}+0.30w_{4}+0.22w_{5}]\geq 1,$$

(10) $$\frac{1}{0.91}[0.83w_{1}+0.28w_{2}+0.58w_{3}+0.66w_{4}+0.33w_{5}]\geq 1,$$

(11) $$\frac{1}{0.91}[1.86w_{1}+1.68w_{2}+1.25w_{3}+1.28w_{4}+1.95w_{5}]\geq 1,$$

(12) $$\frac{1}{0.91}[0.28w_{1}+0.62w_{2}+0.91w_{3}+0.87w_{4}+0.38w_{5}]\geq 1,$$

(13) $$w_{1},\;w_{2},\ldots w_{5}\geq 0.$$

Finally, we incorporated additional judgment constraints appropriate for each weighting scheme. For example, an improved DEA that included EI99 weighting scheme for a DMO applied 3 additional constraints (Eqn. 14–16), in addition to the CDEA constraints (Eqn. 8–13).

(14) $$w_{1}+w_{5}=0.4\times \sum w_{i}.$$

(15) $$w_{2}+w_{4}=0.4\times \sum w_{i}.$$

(16) $$w_{3}=0.2\times \sum w_{i}.$$

All other weighting schemes and corresponding constraints are provided in Supplemental Data.

## RESULTS

The mean-normalized life-cycle costs per cubic meter of RWH ranged from 0.58 to 1.5 (US$/US$) and life cycle blue water use per cubic meter of RWH ranged from 0.1 to 2.0 (m^3^/m^3^) for 20 DMOs (Figure 1, Table 2). A complete description of indicators and the mean-normalized data for 20 DMOs are provided in Supplemental Data. The life cycle costs and life cycle blue water use impacts of 20 DMOs revealed a Tradeoff Line with a potential sustainable solution domain and Optimal EE Point (Figure 1), which included a subset of 5 relatively more sustainable DMO options (DMOs 19, 16, 11, 20, and 5), with DMOs 5, 20, and 11 corresponding to lower life cycle impacts but with higher life-cycle costs than DMO16.

**Figure 2:**
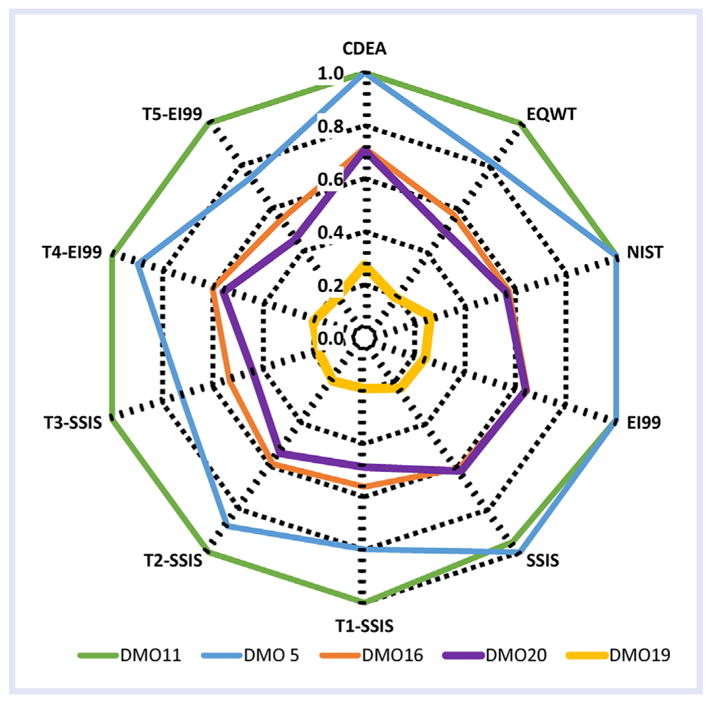
Sensitivity analysis of sustainability scores or modified EE measures of 5 DMOs to 10 weighting schemes depicting the most sustainable DMO11 and least sustainable DMO19 within the defined framework: CDEA, EQWT, NIST, EI99, SSIS, Threshold 1 to SSIS (T1-SSIS) (*w*_1_ = *w*_5_ and *w*_2_ = *w*_4_), Threshold 2 to SSIS (T2-SSIS) (*w*_1_:*w*_5_ = 67%:33%; *w*_2_:*w*_4_ = 67%:33%), Threshold 3 to SSIS (T3-SSIS) (*w*_1_:*w*_5_ = 33%:67%; *w*_2_:*w*_4_ = 33%:67%), Threshold 4 to EI99 (T4-EI99) (*w*_1_:*w*_5_ = 67%:33%; *w*_2_:*w*_4_ = 67%:33%), Threshold 5 to EI99 (T5-EI99) (*w*_1_: *w*_5_ = 33%:67%; *w*_2_:*w*_4_ = 33%:67%). CDEA = classical data envelopment analysis; DMO = decision management objective; EE = eco-efficiency; EI99 = Eco-Invent 99; EQWT = equal weights; = National Institute of Standards and Technology stakeholder panel; SSIS = Sustainable Society Index scheme.

The sustainability scores for the 5 DMOs using the 10 weighting schemes are visualized best as a radar plot (Figure 2; Table 2 for details of DMO notation). The sustainability score attenuated from the outermost wave toward the inner wave of the radar, representing the most-and least-sustainable DMOs. Overall, sustainability scores for the 5 DMOs ranged from 0.18 to 1.0, with the lowest value for DMO19 using the Threshold 5 to EI99. DMO11 received the highest score for all schemes except SSIS, which ranked DMO5 highest. Classical DEA, NIST, and EI99 produced 2 DMOs (DMO5 and DMO11) as the most sustainable, creating a situation of EE nonuniqueness which is a main drawback of CDEA (Sironen et al. 2014). All threshold schemes (Thresholds 1–5) and EQWT overcame the EE nonuniqueness problem, revealing DMO11 as most sustainable. The threshold schemes not only produced improved sustainability scores but also eliminated the possibility of obtaining impractical zero weights (Table 3). Optimal weights of DMO16, DMO19, and DMO20 were zero when no thresholds were implemented. Zero weights can be unrealistic, which means the formulation would have been nonzero if a different scheme were chosen. Zero weights persisted in many of the indicators such as human health cancer and ecotoxicity of DMO19 and DMO20 with NIST scheme; global warming potential and ecotoxicity of DMO16, DMO19, and DMO20 with EI99 scheme; and human health cancer of all DMOs with SSIS scheme (Table 3).

## DISCUSSION

We demonstrated a sustainability analysis methodology using the example of 20 competing RWH system DMOs that differ by material and energy components. The EE Tradeoff Line can be used to explore sustainable solution options among DMOs in consideration. In our example 5 relatively more sustainable options, DMO19, DMO16, DMO11, DMO20, and DMO5, emerged among the 20 when the life cycle costs were compared against life cycle blue water use. Solution domain and optimal EE Point vary with the type of indicators selected and the number of DMOs. A different Tradeoff Line consisting of slightly different DMOs may emerge with alternative indicators; therefore, analysts are recommended to evaluate a Tradeoff Line of various preferred indicators. Also, analysts may prefer an alternate DMO from the Tradeoff Line that best meets preferences for environmental over economic indicators. For example, if avoidance of environmental impact is valued over cost, then DMO5, DMO11, or DMO20 would be preferred. The integration of the indicators provided a single sustainability score, enabling an overall comparison of DMOs.

Threshold weighting schemes were necessary to determine unique sustainability scores because the threshold schemes avoided the sustainability nonuniqueness problem and impractical zero weights. Classical DEA, NIST, EI99, and SSIS schemes resulted in zero weights; therefore, corresponding sustainability scores were nonrealistic. However, the threshold weighting schemes T1-SSIS, T2-SSIS, T3-SSIS, T4-EI99, and T5-EI99 produced unique sustainability scores providing insights into DMO selection. Also, examination of weighting composition, zero and nonzero weights, facilitated the selection of a weighting scheme that avoided zero weights. An analyst can select a specific weighting scheme using the relative weights of indicators, and they can also select a DMO as the best sustainable alternative. For example, for the most sustainable DMO11, global warming potential had the greatest relative environmental weight of 0.89 (NIST) and lowest relative weight of 0.23 (T2-SSIS) to reach the optimal, highest sustainability score of 1.0. Similarly, for DMO5, blue water use had the greatest relative weight of 1.02 (SSIS) and lowest relative weight of 0.41 (T3-SSIS). If blue water use was weighted higher than human health, DMO5, the suboptimal DMO, considering nonzero highest weight to blue water (0.75) and sustainability score of 0.90 with Threshold 4, would be selected. Further, the importance of considering impact threshold weighting schemes was demonstrated by imposing impact-specific threshold constraints to calculate optimal sustainability scores. As an example, a federal agency reducing GHG emissions by Executive Order (Federal Leadership in…2009) could impose higher thresholds to global warming potential impact (such as 67% as applied in the Threshold 5-EI99). Similarly, an agency target of a 26% increase in water use efficiency by 2020 (Federal Leadership in…2009) would impose higher thresholds to blue water use, such as 67% as applied in Threshold 4-EI99.

The method is also applicable to increase the sustainability score of a less-optimal DMO. A sustainability score less than unity indicates potential room for improvement. DMO19 may be improved by focusing on blue water use, energy demand, and global warming potential. In practice, a specific DMO can be improved by selecting alternate material and energy components with lower environmental impacts and life cycle cost. The selection of weights and weighting schemes is arguably subjective, as is the overall sustainability score because that varies with the preferences of stakeholders. Decision making becomes challenging with so many preferences and levels of tradeoffs. Through sensitivity analysis, we attempted to shed light on this limitation and increased transparency with explicit tradeoffs of a system under consideration.

## CONCLUSIONS

The application of threshold weighting schemes is recommended over other schemes for sustainability analysis. Weighting schemes EQWT, T1-SSIS, T2-SSIS, T3-SSIS, T4-EI99, and T5-EI99 resulted in nonzero weights and unique sustainability scores. The weighting schemes CDEA, NIST, and EI99 produced unrealistic zero weights and nonunique scores. Although producing a single sustainability score, SSIS resulted in zero weights as well. It is also important to consider temporal and spatial variation of impacts. Time variation of an economic indicator was partly addressed by incorporating future discounting, and return to scale was neutralized by mean normalization of data in DEA. Sustainability analysis of systems with high temporal variation (such as long vs short service lives) should be done carefully, including the consideration of different design configurations for climates with high temporal variation. We propose the modified EE framework and accompanying methodology as a best practice with no specific policy example in mind. Both are generally applicable to GI and industrial, environmental, and engineered systems. Other indicators and necessary weighting schemes are easily integrated into the modified EE framework and methodology. Sustainability scores are improved by imposing necessary weighting schemes (e.g., a minimum level of thresholds for impact severity) and evaluating optimal weights. The modified framework is more comprehensive than a green rating system such as LEED because it addresses the triple bottom line of people, planet, and profit. The LEED certification is based on water use efficiency, green infrastructure and green building design, and material and energy efficiency credits, with a primary focus on cost savings through energy reduction (USGBC 2013). Our intent was to illustrate a flexible and transparent methodology that addresses the limitations commonly experienced in sustainability analysis.

## Supplementary Material

Supp **Figure S1.** Publications with “eco-efficiency” and “analysis” search terms in the title.
**Figure S2.** Hypothetical eco-efficiency space with 10 solutions, EE Tradeoff Line, and Optimal EE Point.
**Table S1.** Literature on EE approaches organized chronologically by author
**Table S2.** Life cycle assessment impacts, life cycle costs, and mean-normalized data of a domestic rainwater harvesting (RWH) system for Data Envelopment Analysis
**Table S3.** Description of life cycle cost assessment of a domestic rainwater harvesting system, Decision Management Objective 1, as defined in Table S2

## Figures and Tables

**Table 1:** Modified EE framework for water resource management

Indicator	Unit	Data source	Equations of EE measures
Economic indicator (*A_i_*)			
Life cycle costs (*A*_LC_)	US$	Life cycle cost assessment	*E* = *A*_LC_ / *D_i_* (for all *D_i_*)
Return period (*A*_RP_)	Year	Life cycle cost assessment	*E* = *A*_RP_ / *D_i_* (for all *D_i_*)
Net present value benefits (*A*_NB_)	US$	Life cycle cost assessment	*E* = *A*_NB_ / *D_i_* (for all *D_i_*)
Gross domestic product (*A*_GD_)	US$	National databases	*E* = *A*_GD_ / *D_i_* (for all *D_i_*)
Environmental indicator (*D_i_*)			
Acidification (*D*_AC_)	kg H + mole eq	Life cycle assessment	*E* = *A_i_* / *D*_AC_ (for all *A_i_*)
Total ecotoxicity (*D*_ET_)	CTU (F × m^3^ × d kg^−1^ emitted)	Life cycle assessment	*E* = *A_i_* / *D*_ET_ (for all *A_i_*)
Energy demand (*D*_ED_)	MJ	Life cycle assessment	*E* = *A_i_* / *D*_ED_ (for all *A_i_*)
Total eutrophication (*D*_EU_)	kg N eq	Life cycle assessment	*E* = *A_i_* / *D*_EU_ (for all *A_i_*)
Fossil depletion (*D*_FD_)	kg oil eq	Life cycle assessment	*E* = *A_i_* / *D*_FD_ (for all *A_i_*)
Global warming (*D*_GW_)	kg CO_2_ eq	Life cycle assessment	*E* = *A_i_* / *D*_GW_ (for all *A_i_*)
Metal depletion (*D*_MD_)	kg Fe eq	Life cycle assessment	*E* = *A_i_* / *D*_MD_ (for all *A_i_*)
Ozone depletion (*D*_OD_)	kg CFC11 eq	Life cycle assessment	*E* = *A_i_* / *D*_OD_ (for all *A_i_*)
Smog (*D*_SM_)	kg O_3_ eq	Life cycle assessment	*E* = *A_i_* / *D*_SM_ (for all *A_i_*)
Blue water use (*D*_BW_)	m^3^	Life cycle assessment	*E* = *A_i_* / *D*_BW_ (for all *A_i_*)
Hydrologic impact: percent water availability (*C*_HY_)	%	Hydrologic modeling	*E* = *A_i_* / *C*_HY_ (for all *A_i_*)
Social indicator (*S_i_*)			
Total human health toxicity, cancer (*S*_HH_)	CTU (human population/kg chemical emitted)	Life cycle assessment	*E* = *A_i_* / *S*_HH_ (for all *A_i_*)
Total human health toxicity, noncancer (*S*_HN_)	CTU (human population/kg chemical emitted)	Life cycle assessment	*E* = *A_i_* / *S*_HH_ (for all *A_i_*)
Human health criteria air pollutants (*S*_HC_)	kg PM10 eq	Life cycle assessment	*E* = *A_i_* / *S*_HC_ (for all *A_i_*)

*A_i_* = economic indicators; EE = eco-efficiency; *E* = traditional EE measure; CFC = chlorofluorocarbon; CTU = comparative toxic units; *D_i_* = environmental indicators; F = potentially affected fraction of species; PM10 = particulate matter less than l0 μm in diameter; *S_i_* = social indicators.

**Table 2:** Mean-normalized environmental, social, and economic indicators of the 5 domestic RWH DMOs along Tradeoff Line^a^

RWH system design components	DMO	Mean normalized values (dimensionless)					
		Blue water	Ecotoxicity	Energy demand	Global warming potential	Human health, cancer	Life cycle costs
Plastic pipes 60.1 m; PE tank 6.2 m^3^; no pump	DMO5	0.10	0.49	0.52	0.50	0.27	0.91
Reduced distribution pipes only CPVC 23.7 m; concrete tank 6.2 m^3^, no pump	DMO11	0.65	0.17	0.20	0.30	0.22	0.63
Minimal plastic pipes-CPVC 5 m; concrete tank 6.2 m^3^; pump, 2.5% OM	DMO16	0.83	0.28	0.58	0.66	0.33	0.58
Cast iron pipes 60.1 m; concrete tank 6.2 m^3^; no pump; 2.5% OM	DMO19	1.86	1.68	1.25	1.28	1.95	0.83
Plastic pipes 60.1 m; PE tank 6.2 m^3^; pump; 2.5% OM	DMO20	0.28	0.62	0.91	0.87	0.38	0.83

CPVC = chlorinated polyvinyl chloride; DMO = decision management objective; OM = operation and management; PE = polyethylene; RWH = rainwater harvesting.

a Tabulated 5 DMOs represent potential design configurations from 20 various designs of domestic RWH systems based on Ghimire et al. (2014).

**Table 3:** Optimized weight structure of the 10 modified eco-efficiency analysis schemes (*w_i_* = weights)

1. Classical DEA (CDEA)						6. Threshold 1 to SSIS (*w*_1_ = *w*_5_ and *w*_2_ = *w*_4_)					
**DMO**	**Blue** **water**	**Ecotoxicity**	**Energy** **demand**	**Global warming** **potential**	**Human health,** **cancer**	**DMO**	**Blue** **water**	**Ecotoxicity**	**Energy** **demand**	**Global warming** **potential**	**Human health,** **cancer**
DMO5	0.79	0.50	0.56	0.34	0.46	DMO5	0.59	0.54	0.75	0.54	0.59
DMO11	0.28	0.65	0.52	0.43	0.46	DMO11	0.40	0.37	0.52	0.37	0.40
DMO16	0.63	1.05	0.00	0.00	0.00	DMO16	0.37	0.35	0.48	0.35	0.37
DMO19	0.57	0.00	0.00	1.53	0.00	DMO19	0.53	0.49	0.68	0.49	0.53
DMO20	0.88	1.49	0.00	0.00	0.00	DMO20	0.53	0.49	0.68	0.49	0.53
2. Equal weights (EQWT)						7. Threshold 2 to SSIS (w_1_:*w*_5_ = 67%:33%; *w*_2_:*w_4_* = 67%:33%)					
**DMO**	**Blue** **water**	**Ecotoxicity**	**Energy** **demand**	**Global warming** **potential**	**Human health,** **cancer**	**DMO**	**Blue** **water**	**Ecotoxicity**	**Energy** **demand**	**Global warming** **potential**	**Human health,** **cancer**
DMO5	0.59	0.59	0.59	0.59	0.59	DMO5	0.73	0.68	0.70	0.33	0.36
DMO11	0.41	0.41	0.41	0.41	0.41	DMO11	0.51	0.47	0.48	0.23	0.25
DMO16	0.38	0.38	0.38	0.38	0.38	DMO16	0.47	0.43	0.45	0.21	0.23
DMO19	0.54	0.54	0.54	0.54	0.54	DMO19	0.67	0.62	0.64	0.30	0.33
DMO20	0.54	0.54	0.54	0.54	0.54	DMO20	0.66	0.61	0.63	0.30	0.33
3. NIST stakeholder panel (NIST)						8. Threshold 3 to SSIS (*w*_1_:*w*_5_ = 33%:67%; *w*_2_:*w*_4_ = 33%:67%)					
**DMO**	**Blue** **water**	**Ecotoxicity**	**Energy** **demand**	**Global warming** **potential**	**Human health,** **cancer**	**DMO**	**Blue** **water**	**Ecotoxicity**	**Energy** **demand**	**Global warming** **potential**	**Human health,** **cancer**
DMO5	0.77	0.06	0.77	0.77	0.06	DMO5	0.41	0.38	0.80	0.78	0.84
DMO11	0.23	0.23	0.61	0.89	0.23	DMO11	0.29	0.26	0.55	0.53	0.58
DMO16	0.38	0.38	0.38	0.38	0.38	DMO16	0.26	0.24	0.51	0.50	0.54
DMO19	0.69	0.00	0.69	0.79	0.00	DMO19	0.38	0.35	0.73	0.71	0.76
DMO20	0.60	0.00	0.60	0.60	0.60	DMO20	0.37	0.35	0.73	0.70	0.76
4. Eco-Indicator 99 (EI99)						9. Threshold 4 to EI99 (*w*_1_:*w*_5_ = 67%:33%; *w*_2_:*w*_4_ = 67%:33%)					
**DMO**	**Blue** **water**	**Ecotoxicity**	**Energy** **demand**	**Global warming** **potential**	**Human health,** **cancer**	**DMO**	**Blue** **water**	**Ecotoxicity**	**Energy** **demand**	**Global warming** **potential**	**Human health,** **cancer**
DMO5	0.78	0.38	0.51	0.63	0.24	DMO5	0.75	0.75	0.56	0.37	0.37
DMO11	0.44	0.11	0.36	0.62	0.29	DMO11	0.51	0.51	0.38	0.25	0.25
DMO16	0.60	0.67	0.33	0.00	0.07	DMO16	0.48	0.48	0.36	0.23	0.23
DMO19	0.62	0.00	0.45	0.91	0.28	DMO19	0.68	0.68	0.51	0.33	0.33
DMO20	0.84	0.94	0.47	0.00	0.10	DMO20	0.67	0.67	0.50	0.33	0.33
5. Sustainable Society Index scheme (SSIS)						10. Threshold 5 to EI99 (*w*_1_:*w*_5_ = 33%:67%; *w*_2_:*w*_4_ = 33%:67%)					
**DMO**	**Blue** **water**	**Ecotoxicity**	**Energy** **demand**	**Global warming** **potential**	**Human health,** **cancer**	**DMO**	**Blue** **water**	**Ecotoxicity**	**Energy** **demand**	**Global warming** **potential**	**Human health,** **cancer**
DMO5	1.02	0.00	0.65	0.94	0.00	DMO5	0.42	0.42	0.64	0.85	0.85
DMO11	0.71	0.65	0.45	0.00	0.00	DMO11	0.29	0.29	0.44	0.59	0.59
DMO16	0.66	0.60	0.42	0.00	0.00	DMO16	0.27	0.27	0.41	0.54	0.54
DMO19	0.92	0.00	0.59	0.85	0.00	DMO19	0.38	0.38	0.58	0.77	0.77
DMO20	0.93	0.85	0.59	0.00	0.00	DMO20	0.38	0.38	0.57	0.77	0.77

DEA = data envelopment analysis; DMO = decision management objective; = National Institute of Standards and Technology.
