# Supplementary material for: A Modified Eco-Efficiency Framework and Methodology for Advancing the State of Practice of Sustainability Analysis as Applied to Green Infrastructure
Source: Integr Environ Assess Manag. Author manuscript; Available in PMC 2018 Aug 15. (PMC6093199; doi:10.1002/ieam.1928)
Supplement: Supp — Figure S1. Publications with “eco-efficiency” and “analysis” search terms in the title. Figure S2. Hypothetical eco-efficiency space with 10 solutions, EE Tradeoff Line, and Optimal EE Point. Table S1. Literature on EE approaches organized chronologically by author Table S2. Life cycle assessment impacts, life cycle costs, and mean-normalized data of a domestic rainwater harvesting (RWH) system for Data Envelopment Analysis Table S3. Description of life cycle cost assessment of a domestic rainwater harvesting system, Decision Management Objective 1, as defined in Table S2 [file NIHMS983353-supplement-Supp.docx]

**SUPPLEMENTAL DATA**

**A Modified Eco-Efficiency Framework and Methodology for Advancing the State of Practice of Sustainability Analysis as Applied to Green Infrastructure**

Santosh R. Ghimire^†^ and John M. Johnston^*‡^

^†^Oak Ridge Institute for Science and Education (ORISE) Postdoctoral Research Participant, U.S. Environmental Protection Agency, Office of Research and Development, 960 College Station Rd., Athens, 30605 GA, USA; E-Mail: ghimire.santosh@epa.gov

^[[1]](#footnote-1)‡^U.S. Environmental Protection Agency, Office of Research and Development, 960 College Station Rd., Athens, 30605 GA, USA

This section provides a summary of the literature review on eco-efficiency approaches, mean-normalization method, life-cycle assessment and life-cycle cost assessment data, and sensitivity analysis of weighting schemes.

**Literature Review on EE Approaches**

Publication of eco-efficiency (EE) analyses has increased nearly 20-fold over the period 2000-2012 (Figure S1), with varying definitions and selection of EE indicators. For example, [Rattanapan et al. (2012)](#_ENREF_36) considered production quantity as the economic indicator, and material, energy, water consumption, wastewater production, and solid waste production as environmental indicators of rubber glove production in Thailand. [Wursthorn et al. (2011)](#_ENREF_56) defined an indicator as a parameter that describes the phenomenon or environment and presented sales payments as the economic indicator and emissions as the environmental indicator of EE of industry classifications. The United Nations Conference on Trade and Development defined an EE indicator as “the ratio between an environmental and a financial variable” ([UNCTAD 2004](#_ENREF_51)), which differs from Kielenniva et al.’s (2012) use as a “quantifiable variable” such as population density, acidification, water consumption, or cost efficiency. [Kielenniva et al. (2012)](#_ENREF_20) identified gross domestic product (GDP), value added, change in land value, health risk reduction, and willingness-to-pay as potential economic indicators of contaminated soil management in Finland and selected cost-efficiency as most suitable. The Sustainable Society Foundation of Finland and the Econometric and Applied Statistics Unit of European Commission’s Joint Research Centre considered 21 sustainability indicators (food, sanitation, water, education, health, income, energy use, etc.), and clustered them into human, environmental, and economic well-being dimensions in their “Sustainability Society Index” ([Sironen et al. 2014](#_ENREF_46)). The United Nations’ indicators include CO_2_ total emissions per capita (and per $1 GDP), consumption of ozone-depleting substances, and proportion of total water resources used to report progress towards achieving the Millennium Development Goals ([UN 2000](#_ENREF_50)).

Theoretical EE space with an EE Tradeoff Line shows the potential solution domain from which an analyst can identify the Optimal EE Point (Figure 2). The EE Tradeoff Line is similar to the Isoquant curve in economics related to production technical efficiency ([Farrell 1957](#_ENREF_8)). Normalized environmental indicators are aggregated and integrated with normalized economic indicators, with a single modified EE measure utilizing a statistical approach such as Data Envelopment Analysis.


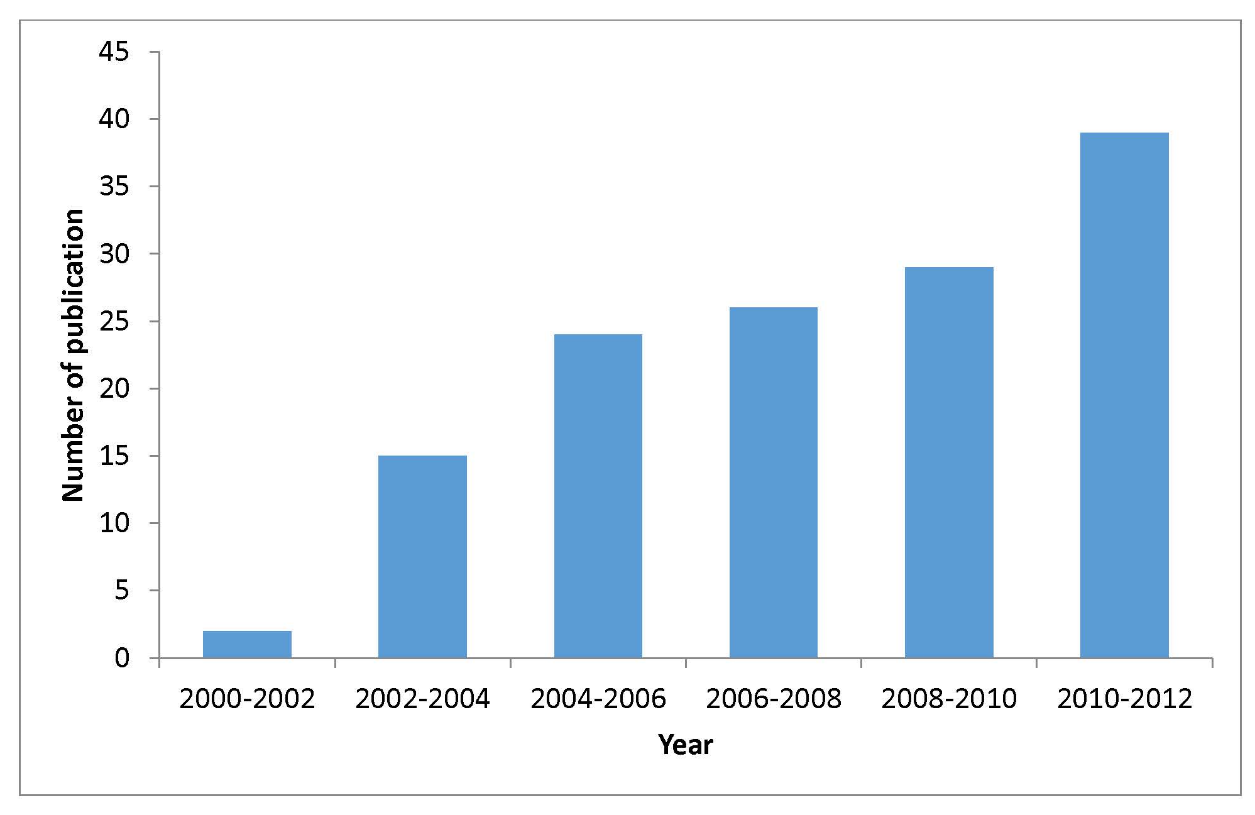


**Figure S1:** Publications with “eco-efficiency” and “analysis” search terms in the title (accessed 2013).


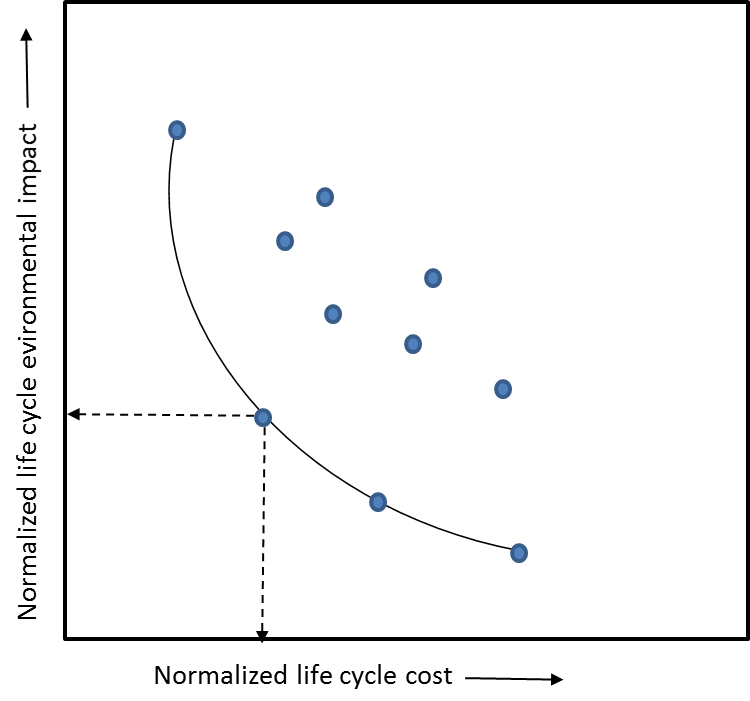


**Figure S2.** Hypothetical eco-efficiency space with 10 solutions, EE Tradeoff Line and Optimal EE Point.

**Table S1:** Literature on EE approaches organized chronologically by author (LCCA = Life-cycle cost assessment, LCA= Life-cycle assessment, DEA = Data Envelopment Analysis)

| **Author** | **a. Application area; b. EE Indicator** | **Model/Theory** |
| --- | --- | --- |
| [Park et al. (2015)](#_ENREF_33) | 1. U.S. manufacturing and transportation modes: air, rail, truck, and water 2. environmental indicators: GHG emissions, energy use, and water withdrawals; economic indicator: economic outputs ($) | Economic Input-Output LCA and  Principal Component Analysis |
| [Meylan et al. (2014)](#_ENREF_29) | 1. Swiss municipal solid waste management 2. environmental indicators: environmental impact increases/reductions; economic indicator: Gross value added gains/losses | LCA and survey based EE analysis |
| [Kamande and Lokina (2013)](#_ENREF_19) | 1. Kenyan manufacturing firms 2. environmental indicators: fuel, water, electricity, and waste; economic indicator: return on assets as profitability | Empirical relationship between the EE and profitability; EE: environmental performance, value per unit environmental effects |
| [Tatari and Kucukvar (2012)](#_ENREF_48) | 1. construction materials 2. environmental indicators: acidification, ecological toxicity, eutrophication, global warming, fossil depletion, smog, water depletion, human health, ozone depletion; economic indicator: life cycle costs | DEA; LCA; Building for Environmental and Economic Sustainability software; EE: ratio of life cycle cost to LCA |
| [Wu et al. (2012)](#_ENREF_55) | 1. urban material metabolism in China 2. environmental indicators: relative increase in resource use (e.g., water and electricity resources) and environmental pollutant discharge (e.g., SO_2_, waste, dust); economic indicator: relative increase in gross domestic production | Positive  Matrix Factorization, a statistical tool for weighting and aggregating indicators; EE: geometric mean of resource efficiency and environmental efficiency |
| [Quariguasi-Frota-Neto and Bloemhof (2012)](#_ENREF_35) | 1. remanufactured desktop computers and mobiles 2. environmental indicator: cumulative energy demand; economic indicator: willingness-to-pay, production costs | LCA of cumulative energy demand; EE: ratio of relative energy consumption to the relative price of remanufacturing |
| [Leal et al. (2012)](#_ENREF_27) | 1. bioethanol transportation in Brazil 2. environmental indicators: energy consumption, CO_2_ emission, and safety (cost of accidents); economic indicator: monetary value (freight revenue) | Survey-based environmental indicator, DEA; EE: ratio of monetary value to environmental influences |
| [Li et al. (2011)](#_ENREF_28) | 1. building manufacturing 2. environmental indicator: emergy (function of material, land, and waste); economic indicator: building space | EE as the ratio of building space to environmental indicator, i.e., emergy |
| [Sanjuan et al. (2011)](#_ENREF_43) | 1. cheese production 2. environmental indicators: global warming, eutrophication, water use; two economic indicators: economic value added and net income | DEA and LCA; EE: ratio of economic  value added to environmental indicator |
| [Picazo-Tadeo et al. (2011)](#_ENREF_34) | 1. rain-fed agricultural system in Spain 2. environmental indicators: nitrogen and phosphorous balances (kg/area), pesticide risk, energy use; economic indicator: value added (sales); and socio-economic indicator: income from farming | DEA; EE: ratio of economic  value added to environmental indicator |
| [Oggioni et al. (2011)](#_ENREF_31) | 1. global cement industries 2. environmental indicators: material and energy as inputs and emissions (e.g., CO_2_) as undesirable output; economic indicators: cement production as desirable output | DEA |
| [Hahn et al. (2010)](#_ENREF_16) | 1. German companies 2. return with a given amount of an environmental resource; opportunity cost of the resource use (function of benchmark EE (e.g., CO_2_-emissions per German national economy) | Ratio of return to opportunity cost, i.e., return-to-cost-ratio |
| [Vercalsteren et al. (2010)](#_ENREF_53) | 1. drinking cups at public events in Belgium 2. environmental indicators: climate change, ozone depletion, ecotoxicity, human toxicity; economic indicators: life cycle cost | LCA and LCCA  EE portfolio (cost versus aggregated environmental indicator) |
| [Chen et al. (2010)](#_ENREF_5) | 1. U.S. electric utilities 2. environmental indicators: SO_2_, CO_2_ undesirables and total sales as outputs; economic indicator: plant value, e.g., electricity purchased (MWh), as input | frontier approach, similar to DEA |
| [Shim and Eo (2010)](#_ENREF_45) | 1. Korean fossil-fueled power plants 2. labor, capacity, and the amount of GHG emissions as inputs and power generation and sales as outputs | DEA |
| [Barba-Gutiérrez et al. (2009)](#_ENREF_2) | 1. household appliances (e.g., a washing machine) 2. environmental indicators: human health, ecosystem quality, and resource depletion; economic indicator: retail price | DEA and LCA |
| [Zhang et al. (2008)](#_ENREF_58) | 1. industrial systems in China 2. environmental indicators: solid waste as undesirable outputs; economic indicators: value added of industries as desirable outputs, and material and energy as inputs | DEA |
| [Borén (2008)](#_ENREF_3) | 1. wastewater treatment plant in Sweden 2. environmental impacts: global warming, acidification, eutrophication; economic indicator: life cycle costs | LCA and LCCA  EE portfolio (cost versus aggregated environmental indicator) |
| [Rüdenauer et al. (2005)](#_ENREF_41) | 1. alternative private laundry washing machines 2. environmental indicators: relative global warming; economic indicator: relative costs | LCA and LCCA  EE portfolio of indicators |
| [Kolsch et al. (2008)](#_ENREF_21) | 1. industries such as biodiesel vs. diesel industry 2. social indicators: e. g., toxicity potential, wages and salaries | socio-EE analysis (‘SEEBALANCE’) method  socio-EE as the fingerprint of social indicators |
| [Hua et al. (2007)](#_ENREF_18) | 1. paper mills in China 2. environmental indicators: biological oxygen demand as input; economic indicator: paper production as outputs | DEA |
| [Papaefthimiou et al. (2007)](#_ENREF_32) | 1. prototype electrochromic windows 2. environmental indicators: CO_2_ and human toxic emissions, energy efficiency; economic indicator: as cost intensity (cost/energy saving) | EE indicator comparison of alternative glazing windows |
| [Yabar and Morioka (2007)](#_ENREF_57) | 1. plastic recycling in eco-towns in Western Japan 2. environmental impacts: global warming potential, energy consumption, resource consumption; economic indicator: costs of plastic recovery and processes | LCA and LCCA  EE portfolio (impacts vs. costs) |
| [Kortelainen and Kuosmanen (2007)](#_ENREF_24) | 1. sports utility vehicles 2. environmental indicator: various emissions (e.g., climate change, acidification); economic indicator: costs that the use of automobiles generates | DEA |
| [Salmi (2007)](#_ENREF_42) | 1. mining industry in the former Soviet Union 2. environmental indicator: emission or material input; economic indicator: net production | Ratio of economic and environmental indicator.  Complex utilization model (similar to industrial symbiosis) in EE analysis |
| [Kuosmanen and Kortelainen (2005)](#_ENREF_25) | 1. road transportation in Finland 2. environmental indicators: climate change, acidification economic indicator: mileage price of transportation | DEA |
| [Kondo and Nakamura (2005)](#_ENREF_22) | 1. water management and recycling in Japan 2. environmental indicator: landfill consumption; economic indicator: GDP | Two EE measures, CO_2_-efficiency and landfill-efficiency |
| [Korhonen and Luptacik (2004)](#_ENREF_23) | 1. power plants in a European country 2. environmental indicators: dust, NO_x_ and SO_2_ as undesirable outputs; economic indicators: electricity generation as desirable output | DEA |
| [Wall-Markowski et al. (2004)](#_ENREF_54) | 1. renewable resource based-Vitamin B2 and polymers 2. environmental indicators: energy use, material use, risk potential, toxicity potential, and emissions; economic indicator: life cycle costs | Ecological fingerprint |
| [Landsiedel and Saling (2002)](#_ENREF_26) | 1. toxicological risks 2. Environmental indicators: energy use, material use, risk potential, toxicity potential, and emissions; economic indicator: overall costs | Ecological fingerprint |


**Mean-Normalization Method**

Methods to increase data homogeneity include mean normalization and principal component analysis ([Adler and Golany 2007](#_ENREF_1); [Sarkis 2007](#_ENREF_44)). LCA and LCCA data were normalized using the mean-normalization method ([Sarkis 2007](#_ENREF_44)):

${X^{*}}_{ij}= \frac{X_{ij}}{\bar{X}_{ij}}$ (S1)

where

${X^{*}}_{ij}$= Mean normalized value of sustainability indicator i for jth Decision Management Objective (DMO)

$X_{ij}=$ Value of sustainability indicator i for jth DMO

$\bar{X}_{ij}=$ Average value of sustainability indicator i for j number of DMOs

[Boussofiane et al. (1991)](#_ENREF_4) recommended the number of DMOs not be fewer than the number of environmental inputs multiplied by the number of economic outputs. Additional recommendations include the number of DMOs ≥ 2 x number of inputs plus outputs ([Golany and Roll 1989](#_ENREF_15)) and that the number of DMOs ≥ 2 x number of inputs x outputs ([Dyson et al. 2001](#_ENREF_7)). [Sarkis (2007)](#_ENREF_44) noted that using fewer indicators will increase the robustness of DEA formulations. LCCA and LCA data of each DMO are provided in Tables S2 and S3.

**Table S2:** Life-cycle assessment impacts, life-cycle costs, and mean-normalized data of a domestic rainwater harvesting (RWH) system for Data Envelopment Analysis; PVC = polyvinyl chloride, CPVC = Chlorinated PVC

| System design components | Decision management objective (DMO) | Blue water Use (m^3^) | Ecotoxicity (CTU) | Energy demand (MJ) | Global warming potential (kg CO2 eq) | Human health, cancer (CTU) | Life cycle costs present value ($/m^3^) |
| --- | --- | --- | --- | --- | --- | --- | --- |
| Plastic pipes 60.1 m: collection 36.4 m & distribution 23.7 m; polyethylene (PE) tank 6.2 m^3^; pump (medium voltage electricity operation of 2.7 m^3^/day capacity) | DMO1 | 1.7E-03 | 1.3E-03 | 1.5E+01 | 9.1E-01 | 3.7E-11 | 6.2 |
| Cast iron pipes: collection & distribution 60.1 m; PE tank 6.2 m^3^; pump | DMO2 | 8.9E-03 | 4.2E-03 | 2.9E+01 | 1.8E+00 | 2.0E-10 | 8.3 |
| Plastic pipes 60.1 m; concrete tank 6.2 m^3^; pump | DMO3 | 5.4E-03 | 1.0E-03 | 1.3E+01 | 8.6E-01 | 3.8E-11 | 5.2 |
| Cast iron pipes 60.1 m; concrete tank 6.2 m^3^; pump | DMO4 | 1.3E-02 | 3.9E-03 | 2.7E+01 | 1.7E+00 | 2.0E-10 | 7.4 |
| Plastic pipes 60.1 m; PE tank 6.2 m^3^; no pump | DMO5 | 6.2E-04 | 1.1E-03 | 8.5E+00 | 5.3E-01 | 2.6E-11 | 5.1 |
| Cast iron pipes 60.1 m; PE tank 6.2 m^3^; no pump | DMO6 | 7.8E-03 | 3.9E-03 | 2.2E+01 | 1.4E+00 | 1.9E-10 | 7.3 |
| Plastic pipes 60.1 m; concrete tank 6.2 m^3^; no pump | DMO7 | 4.3E-03 | 7.7E-04 | 6.6E+00 | 4.8E-01 | 2.7E-11 | 4.2 |
| Cast iron pipes 60.1 m; concrete tank 6.2 m^3^; no pump | DMO8 | 1.1E-02 | 3.6E-03 | 2.0E+01 | 1.3E+00 | 1.9E-10 | 6.4 |
| Reduced distribution pipes only CPVC 23.7 m; concrete tank 6.2 m^3^; pump | DMO9 | 5.1E-03 | 6.2E-04 | 9.7E+00 | 7.0E-01 | 3.2E-11 | 4.6 |
| Reduced distribution pipes only CPVC 23.7 m; PE tank 6.2 m^3^; pump | DMO10 | 1.5E-03 | 9.2E-04 | 1.2E+01 | 7.5E-01 | 3.0E-11 | 5.5 |
| Reduced distribution pipes only CPVC 23.7 m; concrete tank 6.2 m^3^, no pump | DMO11 | 4.0E-03 | 3.6E-04 | 3.3E+00 | 3.2E-01 | 2.1E-11 | 3.5 |
| Plastic pipes 60.1 m; PE tank 6.2 m^3^; with pump-low voltage electricity | DMO12 | 1.8E-03 | 1.4E-03 | 1.5E+01 | 9.4E-01 | 3.8E-11 | 6.2 |
| Cast iron pipes 60.1 m; concrete tank 6.2 m^3^, with pump-low voltage electricity | DMO13 | 1.3E-02 | 4.0E-03 | 2.7E+01 | 1.8E+00 | 2.0E-10 | 7.4 |
| Plastic pipes 60.1 m; concrete tank 6.2 m^3^; with pump-photovoltaic electricity | DMO14 | 4.5E-03 | 1.3E-03 | 9.0E+00 | 5.0E-01 | 2.9E-11 | 5.2 |
| Plastic pipes 60.1 m; concrete tank 6.2 m^3^; with pump-low voltage | DMO15 | 5.5E-03 | 1.1E-03 | 1.3E+01 | 8.9E-01 | 4.0E-11 | 5.2 |
| Minimal plastic pipes-CPVC 5 m; concrete tank 6.2 m^3^; pump, 2.5% operation & management (OM) | DMO16 | 5.1E-03 | 6.1E-04 | 9.5E+00 | 6.9E-01 | 3.2E-11 | 3.3 |
| Cast iron pipes 60.1 m; PE tank 6.2 m^3^; no pump; 2.5% OM | DMO17 | 7.8E-03 | 3.9E-03 | 2.2E+01 | 1.4E+00 | 1.9E-10 | 5.3 |
| Cast iron pipes 60.1 m; PE tank 6.2 m^3^; with pump; 2.5% OM | DMO18 | 8.9E-03 | 4.2E-03 | 2.9E+01 | 1.8E+00 | 2.0E-10 | 6.2 |
| Cast iron pipes 60.1 m; concrete tank 6.2 m^3^; no pump; 2.5% OM | DMO19 | 1.1E-02 | 3.6E-03 | 2.0E+01 | 1.3E+00 | 1.9E-10 | 4.6 |
| Plastic pipes 60.1 m; PE tank 6.2 m^3^; pump; 2.5% OM | DMO20 | 1.7E-03 | 1.3E-03 | 1.5E+01 | 9.1E-01 | 3.7E-11 | 4.6 |
| Mean | Mean | 6.1E-03 | 2.2E-03 | 1.6E+01 | 1.1E+00 | 9.6E-11 | 5.6 |
| **Mean Normalized data set (dimensionless)** | | | | | | | |
| RWH system design components | DMO | Blue water Use | Ecotoxicity | Energy demand | Global warming potential | Human health, cancer | Life cycle costs present value |
| Plastic pipes 60.1 m: collection 36.4 m & distribution 23.7 m; polyethylene (PE) tank 6.2 m^3^; pump (medium voltage electricity operation of 2.7 m^3^/day capacity) | DMO1 | 0.28 | 0.62 | 0.91 | 0.87 | 0.38 | 1.10 |
| Cast iron pipes: collection & distribution 60.1 m; PE tank 6.2 m^3^; pump | DMO2 | 1.45 | 1.94 | 1.76 | 1.70 | 2.04 | 1.49 |
| Plastic pipes 60.1 m; concrete tank 6.2 m^3^; pump | DMO3 | 0.88 | 0.48 | 0.79 | 0.81 | 0.40 | 0.94 |
| Cast iron pipes 60.1 m; concrete tank 6.2 m^3^; pump | DMO4 | 2.04 | 1.80 | 1.64 | 1.64 | 2.06 | 1.33 |
| Plastic pipes 60.1 m; PE tank 6.2 m^3^; no pump | DMO5 | 0.10 | 0.49 | 0.52 | 0.50 | 0.27 | 0.91 |
| Cast iron pipes 60.1 m; PE tank 6.2 m^3^; no pump | DMO6 | 1.27 | 1.82 | 1.37 | 1.33 | 1.93 | 1.30 |
| Plastic pipes 60.1 m; concrete tank 6.2 m^3^; no pump | DMO7 | 0.70 | 0.36 | 0.40 | 0.45 | 0.28 | 0.75 |
| Cast iron pipes 60.1 m; concrete tank 6.2 m^3^; no pump | DMO8 | 1.86 | 1.68 | 1.25 | 1.28 | 1.95 | 1.14 |
| Reduced distribution pipes only CPVC 23.7 m; concrete tank 6.2 m^3^; pump | DMO9 | 0.83 | 0.29 | 0.59 | 0.66 | 0.33 | 0.82 |
| Reduced distribution pipes only CPVC 23.7 m; PE tank 6.2 m^3^; pump | DMO10 | 0.24 | 0.43 | 0.71 | 0.72 | 0.32 | 0.98 |
| Reduced distribution pipes only CPVC 23.7 m; concrete tank 6.2 m^3^, no pump | DMO11 | 0.65 | 0.17 | 0.20 | 0.30 | 0.22 | 0.63 |
| Plastic pipes 60.1 m; PE tank 6.2 m^3^; with pump-low voltage electricity | DMO12 | 0.30 | 0.65 | 0.94 | 0.90 | 0.39 | 1.10 |
| Cast iron pipes 60.1 m; concrete tank 6.2 m^3^, with pump-low voltage electricity | DMO13 | 2.06 | 1.84 | 1.67 | 1.67 | 2.07 | 1.33 |
| Plastic pipes 60.1 m; concrete tank 6.2 m^3^; with pump-photovoltaic electricity | DMO14 | 0.74 | 0.60 | 0.55 | 0.48 | 0.31 | 0.94 |
| Plastic pipes 60.1 m; concrete tank 6.2 m^3^; with pump-low voltage | DMO15 | 0.89 | 0.52 | 0.82 | 0.85 | 0.41 | 0.94 |
| Minimal plastic pipes-CPVC 5 m; concrete tank 6.2 m^3^; pump, 2.5% operation & management (OM) | DMO16 | 0.83 | 0.28 | 0.58 | 0.66 | 0.33 | 0.58 |
| Cast iron pipes 60.1 m; PE tank 6.2 m^3^; no pump; 2.5% OM | DMO17 | 1.27 | 1.82 | 1.37 | 1.33 | 1.93 | 0.95 |
| Cast iron pipes 60.1 m; PE tank 6.2 m^3^; with pump; 2.5% OM | DMO18 | 1.45 | 1.94 | 1.76 | 1.70 | 2.04 | 1.10 |
| Cast iron pipes 60.1 m; concrete tank 6.2 m^3^; no pump; 2.5% OM | DMO19 | 1.86 | 1.68 | 1.25 | 1.28 | 1.95 | 0.83 |
| Plastic pipes 60.1 m; PE tank 6.2 m^3^; pump; 2.5% OM | DMO20 | 0.28 | 0.62 | 0.91 | 0.87 | 0.38 | 0.83 |

The life-cycle impacts were based on per cubic meter water supply consistent with [Ghimire et al. (2014)](#_ENREF_11) (Table S2). Life-cycle costs included operation and maintenance cost at 5% of capital investment cost, consistent with [Ghimire et al. (2012)](#_ENREF_12) and an effective discount rate at 3% ([Fuller and Petersen 1996](#_ENREF_10); [NIST 2013](#_ENREF_30)), unless otherwise indicated. The unit price of cast iron collection pipe, 101.6-mm, assumed $63.3/m; and cast iron distribution pipe, 19-mm, assumed $12.2/m which is the same as CPVC 19 mm pipe, based on [CPFC (2014)](#_ENREF_6). The cost of a 6.2 m^3^ concrete tank, $1,269, was estimated based on [TWDB (2005)](#_ENREF_49). See additional description in Table S3.

**Table S3:** Description of life-cycle cost assessment of a domestic rainwater harvesting system, Decision Management Objective 1, as defined in Table S2. The LCCA of all other DMOs performed based on the components described in Table S2

| Initial investment | Cost, $, (Base Date 2014 ) | 50-yr present value, $ | Reference |
| --- | --- | --- | --- |
| Collection pipe, 101.6 mm-diameter (dia.) PVC pipe, 36 m @ $15.1/m | 549 | 549 | [CPFC (2014)](#_ENREF_6); [Ghimire et al. (2014)](#_ENREF_11) |
| Distribution, 19 mm-diameter (CPVC), 24 m @ $12.2/m | 289 | 289 | [CPFC (2014)](#_ENREF_7); [Ghimire et al. (2014)](#_ENREF_10) |
| Pipe Total, 60 m | 838 | 838 | As compiled |
| Check valve, 1 piece, 19 mm, Polypropylene | 59 | 59 | [Flomatic (2014)](#_ENREF_9) |
| Pump control valve, 1 piece, 19 mm (cast iron and bronze alloy) | 56 | 56 | [Flomatic (2014)](#_ENREF_9) |
| Filter, first flush, 1 piece | 143 | 143 | [Hicks (2008)](#_ENREF_17) |
| Install pipes and gutter | 881 | 881 | [Ghimire et al. (2012)](#_ENREF_12) |
| Tank, polyethylene, 1 unit, 6.2 m3 | 2,007 | 2,007 | [Ghimire et al. (2014)](#_ENREF_11); [SOM (2003)](#_ENREF_47) |
| Pump, 1 unit (0.5 hp) | 477 | 477 | [Ghimire et al. (2012)](#_ENREF_12) |
| Initial investment sub-total, *I* | 4,462 | 4,462 | As compiled |
| Replacements | $ | $ |  |
| Pumps, 3 pieces (replacements occur at the end of 15, 30, and 45 years): see Descriptions of Replacement Costs section. | 3,707 | 846 | As compiled |
| Check valves, 19 mm, Polypropylene, 6 pieces (replacements occur at the end of 7.5, 15, 22.5, 30, 37.5, and 45 years): see Descriptions of Costs section. | 825 | 188 | [Flomatic (2014)](#_ENREF_9); [Roebuck et al. (2011)](#_ENREF_40) |
| Pump control valves, 19 mm (cast iron and bronze alloy), 6 pieces (replacements occur at the end of 7.5, 15, 22.5, 30, 37.5, and 45 years): see Descriptions of Costs section. | 787 | 179 | [Flomatic (2014)](#_ENREF_9); [Roebuck et al. (2011)](#_ENREF_40) |
| Filter, first flush, 4 pieces (replacements occur at the end of 10, 20, 30 and 40 years): see Descriptions of Costs section. | 1,267 | 289 | [Hicks (2008)](#_ENREF_17); [Roebuck et al. (2011)](#_ENREF_40) |
| Replacement costs sub-total, *R* | 6,585 | 1,502 | As compiled |
| Residual value | $ | $ |  |
| Pump at the end of the 50-year (10/15 of a unit) | 1,395 | 318 | [RICS (2014)](#_ENREF_38); [Fuller and Petersen (1996)](#_ENREF_10) |
| Check valve, 19 mm, Polypropylene (2.5/7.5 of a piece at the end of the 50-year) | 86 | 20 | As compiled |
| Pump control valve, 19 mm (cast iron and bronze alloy) (2.5/7.5 of a piece) at the end of the 50-year) | 82 | 19 | As compiled |
| Residual sub-total, *V* | 1,563 | 357 | As compiled |
| Annual costs | $ | $ |  |
| Operation and maintenance costs, 5% of capital investment, I | 223 | 5,741 | [Ghimire et al. (2012)](#_ENREF_12) |
| Pumping energy, 0.49 kWh/m3 @ $0.103/kWh | 2 | 48 | [Ghimire et al. (2014)](#_ENREF_11); [USEIA (2014)](#_ENREF_52) |
| Annual costs sub-total, *A* | 225 | 5,789 | As compiled |
| Life cycle cost (*A_LC_*) = *I + R -V + A* | 9,709 | 11,397 | As compiled |

**LCCA**

The U.S. guidelines for LCCA ([Register 1999](#_ENREF_37)) (Section 707/Page 30860) define life-cycle costs as “the sum of the present values of investment costs, capital costs, installation costs, energy costs, operating costs, maintenance costs, and disposal costs, over the lifetime of the project, product, or measure.”

We simplify this definition with Equation S2:

| $A_{LC}=C_{I}-C_{S}+C_{OM}+C_{E}+C_{R}+C_{D}$ | (S2) |
| --- | --- |

where

A_LC_ = Life-cycle costs of a RWH system

*C_I_* = Total investment costs

*C_S_* = Total salvage value (residuals) of the system at the end of life

*C_OM_* = Operation and maintenance costs

*C_E_* = Energy costs

*C_R_* = Total replacement costs of the system (e.g., pump with shorter service lives are replaced)

*C_D_* = Total disposal costs of the system (assumed to be zero)

To simplify the analysis and to be consistent with other studies, we excluded disposal costs related to decommissioning, disposal and transport of the waste material ([Fuller and Petersen 1996](#_ENREF_10); [Roebuck 2007](#_ENREF_39); [Ghimire et al. 2012](#_ENREF_12)). We acknowledge that disposal costs would be taken into consideration for the LCCA; however, with an analysis period of 50 years, the present value of future disposal costs are small compared to overall costs, even for a major system component such as storage tank ([Roebuck 2007](#_ENREF_39)).

**Replacement Costs**

The present value of each replacement cost (*R_PV_)* was estimated using the corresponding single present value (SPV) discount factor:

***$R_{PV}=F_{t}\text{ x }\left(1+i\right)^{-t}$*** (S3)

where

*(1+i)^-t^* = SPV discount factor, *t* being the service life of domestic RWH system (50 year)

*i* = the real discount rate (0.03) suggested by the National Institute of Standards and Technology ([NIST 2013](#_ENREF_30)).

In Equation S3, we estimated, *F_t_,* the actual future replacement cost of an item, with price escalation rates deviating from general inflation rates at 3%, as suggested by the NIST LCCA handbook. For example, the lump-sum total replacement costs of three unit pumps of service life 15 years may be estimated as:

$F_{t}= {477 x \left( 1+.03 \right)}^{15}+477 x \left( 1+.03 \right)^{30}+477 x \left( 1+.03 \right)^{45}$ *=* $3,707 (S4)

Corresponding present value, *R_PV,_* of all pump replacements in a domestic RWH system may be estimated at $3,707 x (1+0.03)^-50^ = $846.

Present value of replacement costs may also be estimated by reducing the future replacement costs to the base-date at each replacement time, which would result in a lower life-cycle cost than the method used here.

**Pumping Energy Costs**

The annual energy cost of pump operation was estimated utilizing the average electric rate, energy usage per cubic meter water, and the volume of pumped water, as shown in Equation S5:

*C_E_ = P* x *E* x *Q*  (S5)

where

*C_E_* = Annual energy cost, $/year

*P* = Average retail price of electricity, $/kWh ($0.1030/kWh) ([USEIA 2014](#_ENREF_52))

*E* = Annual energy use per cubic meter water supply (0.49 kWh/m^3^) ([Ghimire et al. 2014](#_ENREF_11))

Q = Annual pumped water (37 m^3^/year) ([Ghimire et al. 2014](#_ENREF_11))

Annual pumping energy cost is a function of pumped water volume (water demand), system dynamic head, electricity price, and energy use. These were not included in this analysis because our goal was to demonstrate the sustainability analysis method. For all annual costs, we accounted for the time value of money by estimating the present value (PV) of the annually recurring uniform amounts of a system (e.g., pumping energy costs, operation and maintenance costs), as described by Equation S6.

$C_{PV}=A\text{ x }\frac{\left(1+i\right)^{n}-1}{i\left(1+i\right)^{n}}$ (S6)

where

$C_{PV}$= present value of annual cost

*A* = Annual costs, $

$\frac{\left(1+i\right)^{n}-1}{i\left(1+i\right)^{n}}$ *=*Uniform present value (UPV) factor

*i* = real discount rate (0.03) ([NIST 2013](#_ENREF_30))

*n* = number of compounding periods (years) (i.e., service life = 50 years)

**Residual Value Estimation**

Residual value of a component was estimated by the straight-line method of depreciation recommended by the Royal Institution of Chartered Surveyors and the U.S. Department of Energy ([Fuller and Petersen 1996](#_ENREF_10); [RICS 2014](#_ENREF_38)).

**Weighting Schemes for Sensitivity Analysis**

Weighting schemes and associated constraints representing the subjectivity evaluation are provided below:

1. Classical DEA

The constraints are as shown in Equations (6a-d, article).

1. Equal weights (*w_X_*) for all impact categories

| *w_1_ = w_2 =_….. = w_X_* | (S7) |
| --- | --- |

In our case, *X* =5 and weights are: *w_1_* = Blue water use; *w_2_* = ecotoxicity total, *w_3_* = cumulative energy demand; *w_4_* = global warming potential, and *w_5_* = human health, cancer.

1. National Institute of Standards and Technology’s stakeholder panel: Global warming potential ≥ Energy demand ≥ Blue water use ≥ Human health ≥ Ecotoxicity ([Gloria et al. 2007](#_ENREF_13); [Tatari and Kucukvar 2012](#_ENREF_48)).

| *w_4 ≥_ w_3≥_ w_1≥_ w_5≥_ w_2_* | (S8) |
| --- | --- |
|  |  |

1. Eco-Indicator 99: Human Health - 40%, Ecosystem Quality - 40%, and Resources - 20% ([Goedkoop and Spriensma 2000](#_ENREF_14)). The life cycle impacts were reduced to the three major categories: Human Health or Human well-being = blue water use and human health cancer; Ecosystem Quality or Environmental well-being = ecotoxicity and global warming potential; and Resources = cumulative energy demand.

| *w_1_ + w_5_ =* 0.4 x *∑w_i_* | (S9) |
| --- | --- |
| *w_2_ + w_4_ =* 0.4 x *∑w_i_* | (S10) |
| *w_3_ =* 0.2 x *∑w_i_* | (S11) |

1. Sustainable Society Index scheme: Human well-being- 39%, Environmental well-being - 36%, and Economic well-being - 25%) ([Sironen et al. 2014](#_ENREF_46)). The life-cycle impacts were reduced to major categories of Human well-being or Human Health = blue water use and human health cancer; Environmental well-being or Ecosystem Quality = ecotoxicity and global warming potential; and Economic well-being or Resources = cumulative energy demand. Weights of 0.39, 0.36, and 0.25 were assigned to Human well-being, Environmental well-being, and Economic well-being, respectively, consistent with [Sironen et al. (2014)](#_ENREF_46).

| *w_1_ + w_5_ =* 0.39 x *∑w_i_* | (S12) |
| --- | --- |
| *w_2_ + w_4_ =* 0.36 x *∑w_i_* | (S13) |
| *w_3_ =* 0.25 x *∑ w_i_* | (S14) |

1. Threshold 1 to Sustainable Society Index scheme: Under this scheme, we imposed threshold constraints of equal impact weights for Human well-being and Environmental well-being of SSIS, as defined by:

| *w_1_ = w_5_ =* (0.39/2) x *∑w_i_* | (S15) |
| --- | --- |
| *w_2_ = w_4_ =* (0.36/2) x *∑w_i_* | (S16) |
| *w_3_ =* 0.25 x *∑ w_i_* | (S17) |

1. Threshold 2 to Sustainable Society Index scheme: Under this scheme, we imposed threshold constraints of unequal (67% versus 33%) impact weights for Human well-being and Environmental well-being categories of SSIS, as defined by:

| *w_1_ =* (0.67 x 0.39) x *∑w_i_* | (S18) |
| --- | --- |
| *w_5_ =* (0.33 x 0.39) x *∑w_i_* | (S19) |
| *w_3_ =* 0.25 x *∑ w_i_* | (S20) |
| *w_2_ =* (0.67 x 0.36) x *∑w_i_* | (S21) |
| *w_4_ =* (0.33 x 0.36) x *∑w_i_* | (S22) |

1. Threshold 3 to Sustainable Society Index scheme: Under this scheme, we imposed threshold constraints of the reverse of Threshold 2 (33% versus 67%) impact weights for Human well-being and Environmental well-being categories of SSIS as defined by:

| *w_1_ =* (0.33 x 0.39) x *∑w_i_* | (S23) |
| --- | --- |
| *w_5_ =* (0.67 x 0.39) x *∑w_i_* | (S24) |
| *w_3_ =* 0.25 x *∑ w_i_* | (S25) |
| *w_2_ =* (0.33 x 0.36) x *∑w_i_* | (S26) |
| *w_4_ =* (0.67 x 0.36) x *∑w_i_* | (S27) |

1. Threshold 4 to Eco-Indicator 99: This scheme imposed threshold constraints of unequal (67% versus 33%) impact weights for Human well-being and Environmental well-being categories of EI99 scheme, as defined by:

| *w_1_ =* (0.67 x 0.4) x *∑w_i_* | (S28) |
| --- | --- |
| *w_5_ =* (0.33 x 0.4) x *∑w_i_* | (S29) |
| *w_3_ =* 0.20 x *∑ w_i_* | (S30) |
| *w_2_ =* (0.67 x 0.4) x *∑w_i_* | (S31) |
| *w_4_ =* (0.33 x 0.4) x *∑w_i_* | (S32) |

1. Threshold 5 to Eco-Indicator 99: This scheme imposed threshold constraints of unequal but reverse of Threshold 4 (33% versus 67%) impact weights for Human well-being and Environmental well-being categories of EI99 scheme, as defined by:

| *w_1_ =* (0.33 x 0.4) x *∑w_i_* | (S33) | |  |
| --- | --- | --- | --- |
| *w_5_ =* (0.67 x 0.4) x *∑w_i_* | (S34) | |  |
| *w_3_ =* 0.20 x *∑ w_i_* | (S35) | |  |
| *w_2_ =* (0.33 x 0.4) x *∑w_i_* | (S36) | |  |
| *w_4_ =* (0.67 x 0.4) x *∑w_i_* | (S37) | |  |
|  | |  | |

The constraints imposed on the Eco-Indicator 99, Sustainable Society Index scheme, and associated threshold constraints (Equations S15-S37) reduced impacts to categories of Human Health or Human well-being, Ecosystem Quality or Environmental well-being, and Resource or Economic well-being ([Goedkoop and Spriensma 2000](#_ENREF_14); [Sironen et al. 2014](#_ENREF_46)). It should be noted that these expert judgment weightings are subjective and may not be independent; for example, resource depletion may affect both human health and ecosystem quality. Regardless, we chose the most widely accepted definition of sustainability comprising Human well-being, Environmental well-being, and Economic wellbeing to illustrate the method.

**REFERENCES**

Adler N, Golany B. 2007. PCA-DEA. In: Zhu J, Cook WD, editors. Modeling Data Irregularities and Structural Complexities in Data Envelopment Analysis. New York, NY: Springer. p. 139-153.

Barba-Gutiérrez Y, Adenso-Díaz B, Lozano S. 2009. Eco-efficiency of electric and electronic appliances: a data envelopment analysis (DEA). *Environmental Modeling & Assessment* **14**:439-447.

Borén T. 2008. Methods for aggregation and communication of life cycle inventory data within the framework of eco-efficiency analysis-A case study at Akzo Nobel [Göteborg, Sweden]: Chalmers University of Technology.

Boussofiane A, Dyson RG, Thanassoulis E. 1991. Applied data envelopment analysis. *European Journal of Operational Research* **52**:1-15.

Chen C-MC, Delmas MA, Montes MJ. 2010. Eco-vs. productive efficiency: A new approach to effective and comparative performance analysis. USA: Institute for Social, Behavioral, and Economic Research, University of California, Santa Barbara.

CPFC. Price Lists [Internet]. USA: Charlotte Pipe and Foundry. Available from: <http://www.charlottepipe.com/price_lists.aspx>

Dyson RG, Allen R, Camanho AS, Podinovski VV, Sarrico CS, Shale EA. 2001. Pitfalls and protocols in DEA. *European Journal of Operational Research* **132**:245-259.

Farrell MJ. 1957. The measurement of productive efficiency. *Journal of the Royal Statistical Society. Series A (General)* **120**:253-290.

Flomatic. Flomatic® Valves Price List March 24, 2014 [Internet]. USA: Flomatic Corporation. Available from: <http://www.flomatic.com/>

Fuller S, Petersen S. 1996. Life-cycle costing manual for the federal energy management program, 1995 Edition. *NIST handbook* **135**.

Ghimire SR, Johnston JM, Ingwersen WW, Hawkins TR. 2014. Life Cycle Assessment of Domestic and Agricultural Rainwater Harvesting Systems. *Environmental Science & Technology* **48**:4069-4077.

Ghimire SR, Watkins Jr. DW, Li K. 2012. Life cycle cost assessment of a rain water harvesting system for toilet flushing. *Water Science & Technology: Water Supply* **12**:309-320.

Gloria TP, Lippiatt BC, Cooper J. 2007. Life cycle impact assessment weights to support environmentally preferable purchasing in the United States. *Environmental Science & Technology* **41**:7551-7557.

Goedkoop M, Spriensma R. 2000. The Eco-indicator 99: a damage-oriented method for life cycle impact assessment, methodology report. PRé Consultants, Amersfoort, The Netherlands.

Golany B, Roll Y. 1989. An application procedure for DEA. *Omega* **17**:237-250.

Hahn T, Figge F, Liesen A, Barkemeyer R. 2010. Opportunity cost based analysis of corporate eco-efficiency: A methodology and its application to the CO_2_-efficiency of German companies. *Journal of environmental management* **91**:1997-2007.

Hicks WD. 2008. A Cost-Benefit Analysis of Rainwater Harvesting [Project]. [USA]: Duke University. p. 49.

Hua Z, Bian Y, Liang L. 2007. Eco-efficiency analysis of paper mills along the Huai River: An extended DEA approach. *Omega* **35**:578-587.

Kamande MW, Lokina RB. 2013. Clean Production and Profitability An Eco-efficiency Analysis of Kenyan Manufacturing Firms. *The Journal of Environment & Development* **22**:169-185.

Kielenniva N, Antikainen R, Sorvari J. 2012. Measuring eco-efficiency of contaminated soil management at the regional level. *Journal of environmental management* **109**:179-188.

Kolsch D, Saling P, Kicherer A, Grosse-Sommer A. 2008. How to measure social impacts? A socio-eco-efficiency analysis by the SEEBALANCE® method. *International Journal of Sustainable Development* **11**:1-23.

Kondo Y, Nakamura S. 2005. Waste input–output linear programming model with its application to eco-efficiency analysis. *Economic Systems Research* **17**:393-408.

Korhonen PJ, Luptacik M. 2004. Eco-efficiency analysis of power plants: an extension of data envelopment analysis. *European Journal of Operational Research* **154**:437-446.

Kortelainen M, Kuosmanen T. 2007. Eco-efficiency analysis of consumer durables using absolute shadow prices. *Journal of Productivity Analysis* **28**:57-69.

Kuosmanen T, Kortelainen M. 2005. Measuring Eco-efficiency of Production with Data Envelopment Analysis. *Journal of Industrial Ecology* **9**:59-72.

Landsiedel R, Saling P. 2002. Assessment of toxicological risks for life cycle assessment and eco-efficiency analysis. *The International Journal of Life Cycle Assessment* **7**:261-268.

Leal I, Jr., Almada Garcia P, Almeida D’Agosto M. 2012. A data envelopment analysis approach to choose transport modes based on eco-efficiency. *Environment, Development and Sustainability* **14**:767-781.

Li DZ, Zhu J, Hui ECM, Leung BYP, Li QM. 2011. An emergy analysis-based methodology for eco-efficiency evaluation of building manufacturing. *Ecological Indicators* **11**:1419-1425.

Meylan G, Stauffacher M, Krütli P, Seidl R, Spoerri A. 2014. Identifying Stakeholders’ Views on the Eco‐efficiency Assessment of a Municipal Solid Waste Management System. *Journal of Industrial Ecology*.

NIST. 2013. Energy Price Indices and Discount Factors for Life-Cycle Cost Analysis – 2013 Annual Supplement to NIST Handbook 135 and NBS Special Publication 709. National Institute of Standards and Technology, U.S. Department of Commerce.

Oggioni G, Riccardi R, Toninelli R. 2011. Eco-efficiency of the world cement industry: A data envelopment analysis. *Energy Policy* **39**:2842-2854.

Papaefthimiou S, Syrrakou E, Yianoulis P. 2007. Eco-efficiency analysis of an electrochromic smart window prototype. Quantified Eco-Efficiency. Springer. p. 247-259.

Park YS, Egilmez G, Kucukvar M. 2015. A Novel Life Cycle-based Principal Component Analysis Framework for Eco-efficiency Analysis: Case of the United States Manufacturing and Transportation Nexus. *Journal of Cleaner Production* **92**:327-342.

Picazo-Tadeo AJ, Gómez-Limón JA, Reig-Martínez E. 2011. Assessing farming eco-efficiency: A Data Envelopment Analysis approach. *Journal of environmental management* **92**:1154-1164.

Quariguasi-Frota-Neto J, Bloemhof J. 2012. An Analysis of the Eco-Efficiency of Remanufactured Personal Computers and Mobile Phones. *Production and Operations Management* **21**:101-114.

Rattanapan C, Suksaroj TT, Ounsaneha W. 2012. Development of eco-efficiency indicators for rubber glove product by material flow analysis. *Procedia-Social and Behavioral Sciences* **40**:99-106.

Register F. 1999. The President Executive Order 13123, Greening the government through efficient energy management. Federal Register, Part IV. Presidential Documents, The White House, USA.

RICS. RICS draft guidance note: Life cycle costing [Internet]. The United Kingdom: Royal Institution of Chartered Surveyors Available from: <https://consultations.rics.org/consult.ti/life_cycle_costing/viewCompoundDoc?docid=794580&sessionid=&voteid=&partId=795956>

Roebuck RM. 2007. A Whole Life Costing Approach for Rainwater Harvesting Systems, An investigation into the whole life cost implications of using rainwater harvesting systems for non-potable applications in new-build developments in the UK. [UK]: University of Bradford.

Roebuck RM, Oltean-Dumbrava C, Tait S. 2011. Whole life cost performance of domestic rainwater harvesting systems in the United Kingdom. *Water and Environment Journal* **25**:355-365.

Rüdenauer I, Gensch CO, Grießhammer R, Bunke D. 2005. Integrated environmental and economic assessment of products and processes. *Journal of Industrial Ecology* **9**:105-116.

Salmi O. 2007. Eco-efficiency and industrial symbiosis–a counterfactual analysis of a mining community. *Journal of Cleaner Production* **15**:1696-1705.

Sanjuan N, Ribal J, Clemente G, Fenollosa ML. 2011. Measuring and Improving Eco‐efficiency Using Data Envelopment Analysis. *Journal of Industrial Ecology* **15**:614-628.

Sarkis J. 2007. Preparing your data for DEA. In: J. Z, W. C, editors. Modeling data irregularities and structural complexities in Data Envelopment Analysis. New York, NY: Springer. p. 305-320.

Shim HS, Eo SY. 2010. An Analysis of Eco-Efficiency in Korean Fossil-Fueled Power Plants Using DEA. Zero-Carbon Energy Kyoto 2009. Springer. p. 85-89.

Sironen S, Seppälä J, Leskinen P. 2014. Towards more non-compensatory sustainable society index. *Environment, Development and Sustainability*:1-35.

SOM. 2003. Tanks Section UIP 11. USA: State of Michigan.

Tatari O, Kucukvar M. 2012. Eco-Efficiency of Construction Materials: Data Envelopment Analysis. *Journal of Construction Engineering and Management* **138**:733-741.

TWDB. 2005. The Texas Manual on Rainwater Harvesting. USA: Texas Water Development Board, Austin.

UN. United Nations Millennium Declaration, Official list of MDG indicators [Internet]. Available from: <http://www.un.org/millennium/declaration/ares552e.htm>

UNCTAD. 2004. A Manual for the Preparers and Users of Eco-efficiency Indicators. New York: United Nations: United Nations Conference on Trade Development

USEIA. Electricity [Internet]. USA: U.S. Energy Information Administration (EIA). Available from: <http://www.eia.gov/electricity/data.cfm#sales>

Vercalsteren A, Spirinckx C, Geerken T. 2010. Life cycle assessment and eco-efficiency analysis of drinking cups used at public events. *The International Journal of Life Cycle Assessment* **15**:221-230.

Wall-Markowski CA, Kicherer A, Saling P. 2004. Using eco-efficiency analysis to assess renewable-resource–based technologies. *Environmental progress* **23**:329-333.

Wu J, Wu Z, Holländer R. 2012. The application of Positive Matrix Factorization (PMF) to eco-efficiency analysis. *Journal of environmental management* **98**:11-14.

Wursthorn S, Poganietz W-R, Schebek L. 2011. Economic–environmental monitoring indicators for European countries: A disaggregated sector-based approach for monitoring eco-efficiency. *Ecological Economics* **70**:487-496.

Yabar H, Morioka T. 2007. Eco-efficiency analysis of the plastic recovery systems in Hyogo eco-town project. Quantified Eco-Efficiency. Springer. p. 307-327.

Zhang B, Bi J, Fan Z, Yuan Z, Ge J. 2008. Eco-efficiency analysis of industrial system in China: a data envelopment analysis approach. *Ecological Economics* **68**:306-316.

1. To whom correspondence may be addressed: johnston.johnm@epa.gov, Tel.: +1-706-355-8300; Fax: +1-706-355-8302. [↑](#footnote-ref-1)
